# Food-Insecure Women Eat a Less Diverse Diet in a More Temporally Variable Way: Evidence from the US National Health and Nutrition Examination Survey, 2013-4

**DOI:** 10.1155/2019/7174058

**Published:** 2019-10-01

**Authors:** Daniel Nettle, Melissa Bateson

**Affiliations:** Centre for Behaviour and Evolution & Institute of Neuroscience, Newcastle University, Henry Wellcome Building, Framlington Place, Newcastle NE2 4HH, UK

## Abstract

Food insecurity is associated with high body weight amongst women, but not men, in high-income countries. Previous research using food recalls suggests that the total energy intake of food-insecure women is not elevated, though macronutrient composition may differ from that of food-secure women. There is limited evidence on temporal patterns of food consumption. Here, we used food recalls from women in the 2013-4 cycle of the National Health and Nutrition Examination Survey (NHANES, *n* = 2798) to characterise temporal patterns of food consumption in relation to food insecurity. Compared to the food-secure, food-insecure women had more variable time gaps between eating; ate a smaller and less variable number of distinct foods at a time; were more variable from day to day in their time of first consumption; were more variable from day to day in the number of times they ate; and consumed relatively more carbohydrate, less protein, and less fibre. However, their overall energy intake was no higher. Food-insecure women had higher BMIs (2.25 kg/m^2^), and around 15% of the BMI difference between food-insecure and food-secure women was accounted for by their more variable time gaps between eating, their lower diversity of foods, and their lower fibre consumption. Food insecurity is associated with measureable differences in the temporal pattern of food consumption, and some of these differences shed light on how food-insecure women come to have higher body weights.

## 1. Introduction

Food insecurity (FI)—defined as limited or uncertain access to adequate food—is robustly associated with overweight and obesity amongst women, but not men, in high-income countries [1, 2]. Discussions of why this might be the case suggest that experiencing FI promotes greater overall energy intake, specifically by intensifying food motivation [2–4]. However, the evidence for increased energy intake under FI is less clear than sometimes assumed. For example, a recent review paper [5] cites experimental evidence from birds as showing that individuals “increase their food intake when offered access to food at unpredictable times of day” (p. 4). In fact, neither of the studies cited in this passage [6, 7] report any data on the birds' food intake, only that the birds had higher masses in the uncertain food condition. Bird studies that do record food intake have found that the weight gain in response to uncertain food can occur concomitantly with an increase in energy intake [8, 9], with no change in total energy intake [10], or even whilst total energy intake goes down [11].

In humans, studies based on participant-reported intake, usually in the form of 24 hr food recalls, have generally concluded that total energy intake does not differ systematically between women currently experiencing FI and those who are not [12–14], or in some cases, that the energy intake of food-insecure women is less [15]. Studies measuring consumption in ways that bypass participant report, by contrast, find that individuals classified as food-insecure consume more calories when given the opportunity to do so. Nettle et al. [4] gave participants a standardized laboratory “taste test” of snack foods. They found that women who had recently experienced FI (as assessed by a questionnaire prior to the study) consumed more calories, though the association was only statistically significant with one of two measures of FI used. Stinson et al. [16] asked participants to stay in a residential facility and, over 3 days, forage *ad libitum* from vending machines with diverse foodstuffs available. Participants classified as food-insecure at baseline ate around 700 kcal per day more than food-secure participants. These two styles of study—the first measuring consumption in the course of ordinary daily life of food that individuals have to purchase for themselves, the second measuring the response to the sudden, short-term availability of free food—may simply capture different processes. The first suffers from the possibility of imprecise or biased reporting, whilst the second suffers from the possibility that the artificial context of the study does not reflect consumption patterns in everyday life. At present, it is fair to say that the issue of whether experiencing FI leads to increases in energy intake outside of experimental situations is not settled.

However, the food consumption of women experiencing FI may differ in other ways than just total energy intake. Using data from the National Health and Nutrition Examination Study (NHANES), Bergmans et al. [12] found that FI was associated with greater carbohydrate and lesser fibre consumption. Likewise, a number of other studies have found that FI is associated with reduced consumption of fruit, vegetables, and dairy products [17]. Even macronutrient composition does not exhaust the potentially relevant features of a pattern of food intake. For example, experimental studies have shown that the same diet consumed in a temporally irregular rather than a regular pattern causes a lower thermic effect of food [18, 19]. Since the thermic effect of food is a component of energy expenditure, this has implications for weight gain, although participants did not significantly gain weight within the relatively short experimental period of the studies. Correlational evidence suggests that eating fewer meals in the day, and skipping breakfast, are associated with obesity, even after controlling for total energy intake and energetic expenditure from physical activity [20].

This evidence raises the possibility that FI may be associated with subtle changes in the temporal pattern of food intake, even if not the total amount, and this may be relevant to the high body weights observed in women who experience FI. There has been a small amount of prior research on this question. Zizza et al. [14], using NHANES data, found that FI was associated with a reduced number of meals in the day and consequently larger meal size. However, Zizza et al. did not measure *variability* in the timing of food consumption. In evolutionary models of energy regulation, it is variability in the timing of food access that is predicted to trigger fat storage as a buffer against temporary shortfall [2, 21, 22]. Moreover, it is variability in the timing of meals, rather than the number of meals per se, that has been shown to reduce energy expenditure via the thermic effect of food [18, 19]. A further limitation of the study by Zizza et al. [14] is that they did not explore whether the observed differences in food-consumption pattern between food-insecure and food-secure individuals mediated the association between FI and body mass index (BMI). Detecting such mediation would be consistent with differences in the temporal pattern of food consumption being not merely correlates of FI, but playing some causal role in the weight gain of women who experience FI.

Here, we investigated in detail the 24 hr food-consumption recalls of adult women in the 2013-4 cycle of NHANES. Like previous studies, we extracted variables concerning total energy intake, macronutrient composition, and number of eating occasions in the day. Going beyond previous research, we characterised variability over time within each food recall. Temporal variability is of two kinds: intraday (for example, the variation in time gap or energy intake between the meals of a day) and interday (for example, eating more, or more often, on some days than other days). Having developed our set of variables characterising patterns of food consumption, we tested which ones differed between individuals who did and did not report recent experience of FI, both with and without adjustment for sociodemographic characteristics. We then went on to test whether any of the variables that differed by FI status were significant statistical mediators of the FI-BMI relationship. Our general predictions were that, compared to food security, FI would be associated with no greater total energy intake, but greater reliance on carbohydrate and less consumption of fibre; fewer meals in the day; greater intra- and interday variability in consumption pattern; and a later time of first consumption. We focussed on the women, as it is only in women that an association between FI and body weight is found. We report the parallel analyses for the men in the Supplementary Materials. Those analyses may shed light on why the FI-body weight association is lacking in men.

## 2. Materials and Methods

### 2.1. The NHANES Survey

NHANES is an ongoing multistage survey administered by the National Center for Health Statistics. In each two-year cycle, a large diverse sample of the noninstitutionalized US population is recruited to complete a number of questionnaire and examination measures. The sample can be made nationally representative by the application of sampling weights, as is done here (unweighted results are essentially identical). For our main analysis, we selected all adult (18+ years) participants from the 2013-4 cycle who had completed the questionnaire measures and physical examination (*n* = 5924) and then restricted to female gender (*n* = 3101). Of these women, 2798 had at least one day of 24 hr food recall data. Hence, this is the sample size for analyses involving consumption variables.

### 2.2. Study Variables Other Than Food Consumption

FI was measured using the adult questions of the standard USDA questionnaire [23]. This produced a continuous score of 0–10, which can also be categorised using a four-level severity classification. However, 68% of participants scored zero, making them fully food-secure, with the remaining 32% roughly evenly distributed across the other three levels. Using the finer four-level classification would thus reduce statistical power to detect group differences. A recent meta-analysis found no evidence that more severe FI is more strongly associated with obesity or overweight than less severe FI; the consistent weight difference is between women classified as food-secure and those classified as having any degree of food insecurity [2]. Hence, and in line with previous related studies [13], we divided participants into two groups: food-secure (score of 0; *n* = 1925) and food-insecure (score >0; *n* = 892). In the Supplementary Materials, [Supplementary-material supplementary-material-1], for all significant differences we had found using the dichotomous classification, we repeated the analyses using the four-level classification, also examining whether there was evidence of a gradient with increasing severity of FI. Other sociodemographic variables and BMI were captured during the questionnaire and physical examination sessions.

### 2.3. Food-Consumption Variables Derived from 24-hr Food Recalls

Participants completed two separate food recall interviews, the first in person and the second by telephone. Each recall concerned consumption over the 24 hours of the day prior to the interview. The time between the two recall days was 3–10 days. Where appropriate, we averaged the two recall days for participants with both days complete (*n* = 2539). For the remaining 259 participants, variables were based on just one day. In over 99% of cases, day 2 was on a different day of the week to day 1. Thus, pooling the two days helped smooth variability due to day of week.

We extracted variables algorithmically from the food recall files. Foods and beverages consumed are structured in the recall files by consumption event (CE), each CE representing a unique time in the day when something was consumed.  Table 1 defines the key variables extracted. The relative carbohydrate, protein, fat, and fibre variables are residuals from regressions of grams of that particular macronutrient consumed on total grams of food consumed. Thus, they represent the amount of each macronutrient consumed, adjusted for that individual's total food consumption, and hence are all uncorrelated with total energy intake. The calculation of residuals was done on the data from both genders combined. Thus, the means for the women are not exactly equal to zero. The interday difference (IDD) variables are missing for the participants with only one day of food recall data. These variables are based on unsigned values; that is, they are positive regardless of whether day 2 was greater than day 1 or vice versa.

We did not include variables that were completely predicted by combinations of other variables. For example, the mean time gap between CEs is completely predicted by the time of first CE and the number of further CEs in the day. Hence, it was not necessary to include it separately in the set of variables.

### 2.4. Data Analysis Strategy

For our main analyses, we used multivariate analyses of variance (MANOVAs) to examine whether food-secure and food-insecure women differed on each of three sets of food-consumption variables. The sets of variables were: consumption amounts (5 variables concerning total energy intake and macronutrient composition); intraday pattern (6 variables concerning diversity of foods and variability of consumption within a day); and interday variability (5 variables concerning how the two recall days differed from one another). For each set of outcome variables, we performed both a simple and an adjusted MANOVA. For the simple MANOVAs, the sole predictor was FI. For the adjusted MANOVAs, we additionally included control variables: age (years), income (% of federal poverty line, NHANES variable INDFMPIR), education (NHANES variable DMDEDUC2), ethnicity (NHANES variable RIDRETH1), and presence of children in the household (from NHANES variables DMDHHSZA and DMDHHSZB). To follow up significant MANOVA results and understand which variables in each set were driving any overall differences, we then performed univariate general linear models on each outcome variable separately.

Having established which food-consumption variables were significantly predicted by FI after adjustment, we then tested whether any of them predicted BMI, adjusting for income, age, education, and ethnicity. Variables that were both predicted by FI and predicted BMI were considered candidate mediators of the FI-BMI association. To test the extent of mediation, we used R package “lavaan” [24] to estimate how much of the FI-BMI association operated via the potentially mediating food-consumption variables we had identified. We also conducted parallel analyses for the male participants, which are reported in the Supplementary Materials, [Supplementary-material supplementary-material-1]. All analyses were conducted in R [25].

## 3. Results

### 3.1. Descriptive Statistics

Descriptive statistics for the main food-consumption variables are shown in the final column of Table 1.

### 3.2. Main Analyses

Key results are summarised in Table 2. For the set of five consumption amount variables in the unadjusted analysis, there was a significant effect of FI overall. This was driven by food-insecure women consuming relatively more carbohydrate, less protein, less fat, and less fibre than food-secure women. Total energy intake did not differ between food-secure and food-insecure women. In the adjusted analysis, the overall significant difference by FI persisted, though the associations with the individual variables were substantially attenuated. The variables that remained significantly different between food-secure and food-insecure women after adjustment were relative consumption of carbohydrate, protein, and fibre. These three variables also differed significantly with FI status using the four-level classification of FI. Each variable showed a gradient of severity, with the most extreme mean in the severest FI group (see Supplementary [Supplementary-material supplementary-material-1]).

For the six variables concerning intraday patterning of consumption, there was a significant difference between the food-secure and food-insecure women overall in the unadjusted analysis. This was driven by food-insecure women having their first CE later; having fewer CEs in the day; fewer distinct foods per CE; a less variable number of distinct foods per CE; more variable time gaps between CEs; and more variability in energy per CE. The overall significant difference between food-secure and food-insecure women persisted in the adjusted analysis. Amongst the individual variables, the differences in time of first CE, number of CEs, and variability in energy per CE were attenuated to the point of nonsignificance by the adjustment. Thus, after adjustment, significant differences between food-insecure and food-secure women persisted in the mean and variability of foods per CE and the variability of the time gap between CEs. These three variables also differed significantly with FI status using the four-level classification of FI, again showing gradients of severity, with the severest FI producing the most extreme means (Supplementary [Supplementary-material supplementary-material-1]).

For the variables based on interday differences in pattern, the effect of FI in the MANOVA was significant both adjusted and unadjusted. In the unadjusted analysis, food-insecure women differed from food-secure women by having greater interday difference in total energy intake; greater interday difference in the time of the first CE; greater interday difference in the number of CEs; and greater interday difference in the mean time gap between CEs. After adjustment, only the interday difference in the time of the first CE and the interday difference in the number of CEs remained significantly associated with food insecurity. Both of these associations remained significant using the four-level classification of FI, though they lacked clear evidence of severity gradients (Supplementary [Supplementary-material supplementary-material-1]).

**Figure 1:**
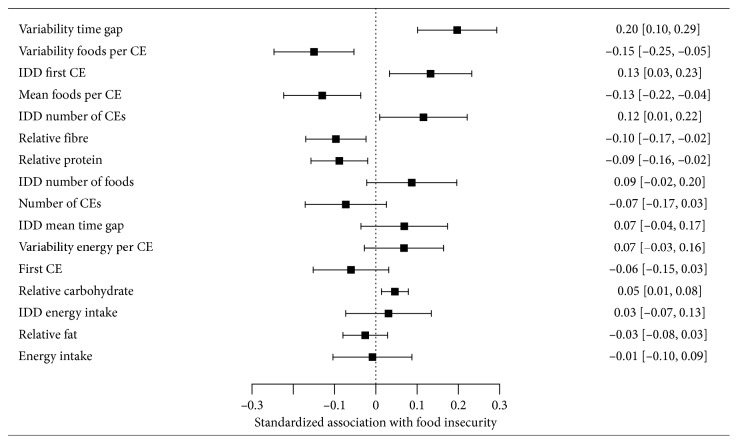
Forest plot of standardized associations between food insecurity status and food consumption variables for NHANES women after adjustment for age, income, education, ethnicity, and presence of children in the household. Variables are sorted so that those more strongly associated with food insecurity status appear higher on the figure. A negative value indicates that food-insecure women have a lower value of the parameter, and a positive value a higher value. Whiskers represent 95% confidence intervals. CE: consumption event. IDD: interday difference (for participants with two separate days of recall data).

To visualize the results and establish which variables were most strongly associated with FI, we standardized parameter estimates from all of the adjusted univariate analyses and produced a forest plot (Figure 1). Variables are sorted by (unsigned) size of the parameter estimate, so that variables more strongly associated with FI appear higher on the figure.

### 3.3. Mediation of the FI-BMI Association

Food-insecure women had higher BMIs than food-secure women (insecure: mean 31.13, sd 8.86; secure: mean 28.77, sd 7.37). This constituted a significant difference after adjustment for income, education, ethnicity, age, and presence of children in the household (*B* = 1.50, se 0.38, *p* < 0.001).

We explored whether the food-consumption variables we had identified as robustly associated with FI could serve as mediators of the association between FI and BMI. We ran models testing whether each of the eight variables with parameter estimates significantly different from zero in Figure 1 predicted BMI, after adjustment for age, income, education, ethnicity, and presence of children in the household (Table 3). Three of the variables (variability time gap, mean foods per CE, and relative fibre consumption) significantly predicted BMI, after adjustment, in the correct direction to serve as a potential mediator. Relative protein consumption also marginally significantly predicted BMI, but in the wrong direction to mediate the FI-BMI association (higher protein, higher BMI).

We then created a multiple mediation model with BMI as the outcome, FI as the predictor, and variability time gap, mean foods per CE, and relative fibre consumption as the mediators. There was an overall positive effect of FI on BMI (total effect 2.21, se 0.33, *z* = 6.77, *p* < 0.001). The pathways via the three mediators accounted for 14.5% of the effect of FI on BMI. This was respectively composed of 4.2% via variability time gap (*z* = 2.17, *p*=0.03), 2.9% via mean foods per CE (*z* = 1.04, *p*=0.30), and 7.4% via fibre consumption (*z* = 3.33, *p*=0.01).

## 4. Discussion

Using 24 hr food recalls from participants in the large, nationally representative NHANES survey, we found that total energy intake was no higher in women classified as food-insecure than in women classified as food-secure. However, patterns of food consumption differed in many other ways. Specifically, food-insecure women had more variable time gaps between eating; ate a smaller and less variable number of distinct foods at a given consumption event; were more variable from day to day in their time of first consumption in the day; were more variable from day to day in the number of times they ate; and consumed relatively more carbohydrate, less protein, and less fibre. These differences were robust to control for age, income, education, ethnicity, and the presence of children in the household. Moreover, we showed in supplementary analyses that most of these variables exhibit clear gradients of severity when FI is divided up into finer categories. Thus, food-insecure women eat a diet that is less diverse than that of food-secure women, but do so in a more temporally variable way. We found that three of the food-consumption differences between food-insecure and food-secure women—their more variable time gaps between eating, their lower number of distinct foods per consumption event, and their lower fibre consumption—partially accounted for their greater body masses.

These findings are informative on several different levels. At the simplest level, they can be seen as a validation of the FI questionnaire measure. We can detect, in the detailed food recalls, that women classified as food-insecure had more variable gaps between meals and relied on a smaller number of foods (and these differences became larger as the severity of their FI increased). The relatively high carbohydrate composition and low protein and fibre composition of food-insecure women suggest a reliance on cheap sources of calories and low consumption of vegetables, fruit, and dairy. This pattern would be expected where budgets for obtaining food are highly constrained [26] and is consistent with previous studies of FI [12, 17]. Our findings suggest that when food-insecure women responded in the affirmative to the FI questions, they were not, overall, just misremembering, exaggerating, or interpreting the question content idiosyncratically. Their dietary behaviour was systematically different from those who did not respond in the affirmative to the FI questions, even after adjusting for their different sociodemographic characteristics, in ways that made sense given the content of the questionnaire items. The food recalls were self-reported too, of course, but represent a very different kind of measure from the general statements of the FI questionnaire.

More deeply, the findings bear on the question of how experiencing FI may lead to high body weight in women in developed countries. The results here concur with those of similar investigations [12–14] in that food-insecure women did not appear to have higher total energy intake. Stinson et al. [16] suggest that the failure to find excess energy intake in food-insecure women in dietary recall studies is to do with the limitations of participant report. For this explanation to be correct, there would have to be not just biased reporting, but differentially biased reporting by FI status in self-report consumption measures. This is plausible, since differential under-reporting of energy intake has been reported in obese individuals [27] and individuals of lower socioeconomic status [28]. However, the recall data used here were adequate to identify numerous other significant differences between the food-consumption patterns of food-insecure and food-secure individuals, spanning from what they ate to when they ate it. It is unclear why participant-recalled data would be adequate to reveal all these other differences, but uniquely inadequate to reveal differences in total energy intake. An alternative possibility is that typical total energy intake is really no higher in women who experience FI. In this case, the increased consumption observed in staged eating opportunities [4, 16], though real, might represent a short-term response to free food amongst people used to being highly constrained in what they can procure.

If total energy intake does not increase in response to experiencing FI, this would not undermine the general principle that weight gain is an adaptive response to FI [21, 22]. In fact, our findings partly justify the linkage made by Nettle et al. [2] between the human FI literature and experimental studies of uncertain food access in animals [6, 29–31]. In the animal experiments, uncertain food access is usually operationalised as variable time gaps between accesses to food, which is shown to lead to mass gain, sometimes without any concomitant increase in total energy intake [10]. The present study is the first to show that food-insecure people—as measured by the conventional human FI questionnaire measure—also experience more variable time gaps between food consumption. Though food-insecure women also differ from food-secure in *what* they eat, the variable that showed the most marked difference between food-insecure and food-secure women in Figure 1 was the variability in *when* they eat it. This is very close to the variable that is experimentally manipulated in the animal studies. This suggests that FI as it is studied in the social sciences is indeed a related phenomenon to the uncertain food access studied in behavioural ecology.

Although we found some support for the contention that differences in food-consumption patterns statistically mediate the association between FI and body weight in women, the extent of the mediation was weak. Between them, the three mediating variables accounted for less than 15% of the association between FI and body weight. At face value, this implies we largely failed to identify what it is that makes women who experience FI become heavier than those who do not. However, the mediation pathways we identified may be more important than the 15% figure suggests.

The reasons for this relate to sources of measurement error in the design, beyond the reliance of the dietary recalls on participant report. Measurement error generally leads to underestimation of associations and may well have this effect in the current study. The FI questionnaire asked about experiences of FI in the last 12 months. Positive responses to items on the questionnaire thus indicate that FI had been experienced recently, but not necessarily that it was still being experienced on the days of the dietary recalls. We can be confident that our “food-secure” group was not experiencing FI on the day of the recalls (since they had not experienced FI at all within the last 12 months), but our “food-insecure” group probably consisted of a mixture of people who were currently experiencing FI and those who had experienced FI, but whose situations had recently improved. In this respect, we believe our design to be conservative. The true differences between average dietary behaviour of people currently being affected by FI and those currently not must be at least as large, if not larger, than those observed between our “food-secure” and “food-insecure” groups.

Second, food-consumption variables here are based on a maximum of two days of data for each participant. Given we have a lot of replication between individuals, then as long as the recall days are a fairly random sample of all days, this still allows high power to detect systematic patterns of difference in the distribution of dietary behaviours between people experiencing FI and those who are not. However, the sampling variability of which days the recalls happened to fall upon is an additional source of noise and hence measurement error. This is more of an issue for the interday difference variables, for which there is no within-person replication, than for the within-day variables, for which there is one within-person replicate.

Given these measurement-error issues, we would expect the measured associations between FI and food-consumption variability, and also between food-consumption variability and body weight, to be underestimates of the true associations. Viewed in this light, rather than seeing it as a shortcoming that we can only account for 15% of the FI-body weight relationship, we find it noteworthy that from just two days of food recalls, we can detect numerous significant differences between women who do and do not report recent experience of FI, and that some of these statistically mediate any of the excess body weight associated with FI.

Nonetheless, there are likely to be important differences between food-insecure and food-secure women not captured in our set of food-consumption variables. We have included no measures relating to physical activity. Though a physical activity questionnaire was administered in this cycle of NHANES, the estimation of metabolic equivalents from the questionnaire responses is indirect. Moreover, physical activity estimated from similar questionnaires is generally poorly correlated with physical activity as objectively measured using accelerometers [32]. Thus, we decided not to attempt estimates of physical activity within this study. It is, however, plausible that FI leads to reductions in physical activity and hence energy expenditure [33]. The evidence for this in humans is currently sparse, though there are suggestive data from animal experiments [31, 34, 35].

Change in energy intake and change in physical activity do not exhaust the possible pathways through which FI could lead to weight gain. Birds, for example, are able to change both their digestive efficiency and overnight metabolic rate in response to changes in food availability [36]. Similar possibilities for humans facing FI have not yet been investigated. An experimental study in humans in which participants were assigned to isocaloric diets that involved either temporally regular or temporally irregular intake found that irregularity reduced energy expenditure via a diminished thermic effect of food [18, 19]. This mechanism could potentially explain why we found evidence for a mediating role of temporal consumption irregularity in the FI-body weight relationship, although the NHANES data do not allow us to test this directly as they include no measure of the thermic effect of food.

Our findings do not provide a clear picture of why FI leads to high body weight in women but not men. Patterns of food consumption differed between food-insecure and food-secure individuals among men in very similar ways to women (see Supplementary Materials, [Supplementary-material supplementary-material-1]). The differences were of similar magnitude (across our 16 variables, the mean of the unsigned standardized effect size for the food-insecure to food-secure comparison was 0.09 for the women and 0.07 for the men). Moreover, food-consumption variables predicted body weight amongst men in much the same way they did amongst women (see Supplementary Materials, [Supplementary-material supplementary-material-1]). The only notable difference was in the case of the variability in time gap between CEs. In women, a greater variability in time gap was clearly associated with a higher BMI. In men, the corresponding association was null. Interestingly, the experimental findings on the metabolic effects of irregular versus regular meal pattern have all been from studies of solely female participants [18, 19, 37]. Thus, it is possible that women respond physiologically to variability in the time gap between food intake in a way that men do not. However, this effectively restates the sex difference in the response to FI via a different variable: it does not, in itself, explain that sex difference.

## 5. Conclusions

In a large, nationally representative US sample, we have shown that experience of FI, as measured by the USDA questionnaire, corresponds to measurably different patterns of food consumption. In line with previous studies, we found that food-insecure women eat more carbohydrate and less protein and fibre, but appear to consume the same amount of energy overall. We also showed that they ate a lower diversity of foods, and, critically, that they showed greater temporal variability in their intake. These variations in food-consumption patterns may be part of the reason that women who experience FI end up with higher body weights.

## Figures and Tables

**Table 1:** Variables extracted from the food recalls.

	Variable name	Definition	Units	Women's mean (sd)
Consumption amounts	Energy intake	Total energy intake per day	kcals	1779 (704)
	Relative carbohydrate	Relative carbohydrate	g	2.61 (30.20)
	Relative protein	Relative protein	g	−3.26 (19.93)
	Relative fat	Relative fat	g	0.43 (18.76)
	Relative fibre	Relative fibre	g	0.23 (6.60)

Intraday pattern	First CE	Time of first CE	Hours from midnight	7.93 (2.27)
	Number of CE	Number of CEs per day	Number	5.57 (1.63)
	Mean foods per CE	Mean number of distinct foods per CE	Number	9.68 (3.28)
	Variability foods per CE	Intraday standard deviation number of distinct foods per CE	Number	5.42 (1.93)
	Variability time gap	Intraday standard deviation in time gap between CEs	Minutes	104.27 (48.79)
	Variability energy per CE	Intraday standard deviation Kcals per CE	kcals	322.2 (152.65)

Interday variability (participants with 2 days of data)	IDD energy intake	Interday difference in energy intake	kcals	627.91 (577.70)
	IDD first CE	Interday difference in time of first CE	Hours	1.65 (2.15)
	IDD number of foods	Interday difference in number of foods	Number	4.61 (3.84)
	IDD number of CEs	Interday difference in number of CEs	Number	1.48 (1.32)
	IDD mean time gap	Interday difference in mean time gap between CEs	Minutes	63.42 (70.67)

CE: consumption event. IDD: interday differences (for participants with two separate days of food recall data).

**Table 2:** Parameter estimates for the difference between food-secure and food-insecure women. Adjusted models include income, education, ethnicity, having children in the household, and age as additional predictors. Food-secure is the reference category, and hence the parameter estimates represent the deviation of food-insecure women from the food-secure mean.

	Unadjusted		Adjusted	
	*B* (se)	*p*-value	*B* (se)	*p* value
*Consumption variables*	MANOVA F(5, 2792) = 21.52	<0.001	MANOVA F(5, 2579) = 36.32	<0.001
Energy intake	6.74 (29.55)	0.82	−5.79 (34.33)	0.87
Relative carbohydrate	9.92 (1.34)	<0.001	4.30 (1.55)	0.006
Relative protein	−4.50 (0.99)	<0.001	−2.63 (1.04)	0.01
Relative fat	−3.22 (0.81)	<0.001	−0.88 (0.94)	0.35
Relative fibre	−2.02 (0.28)	<0.001	−0.80 (0.31)	0.01

*Intraday pattern variables*	MANOVA F(6, 2685) = 24.68	<0.001	MANOVA F(6, 2482) = 27.67	<0.001
First CE	0.25 (0.09)	0.007	−0.14 (0.11)	0.20
Number of CEs	−0.50 (0.07)	<0.001	−0.12 (0.08)	0.15
Mean foods per CE	−1.49 (0.14)	<0.001	−0.43 (0.16)	0.006
Variability foods per CE	−0.90 (0.09)	<0.001	−0.29 (0.10)	0.002
Variability time gap	16.15 (2.07)	<0.001	9.61 (2.39)	<0.001
Variability energy per CE	22.07 (6.48)	<0.001	10.40 (7.47)	0.16

*Interday variability variables*	MANOVA F(5, 2516) = 8.96	<0.001	MANOVA F(5, 2327) = 9.31	<0.001
IDD energy intake	65.15 (25.91)	0.01	17.77 (30.51)	0.56
IDD first CE	0.52 (0.09)	<0.001	0.28 (0.11)	0.01
IDD number of foods	0.05 (0.18)	0.79	0.33 (0.21)	0.12
IDD number of CEs	0.11 (0.06)	0.06	0.15 (0.07)	0.03
IDD mean time gap	13.32 (3.26)	<0.001	4.87 (3.78)	0.20

**Table 3:** Results of models testing whether each of the food consumption variables significantly associated with food insecurity predicts body mass index in NHANES women. All models are adjusted for age, income, education, ethnicity, and presence of children in the household.

Predictor	*B* (se)	*p* value
Relative carbohydrate	−0.01 (0.005)	0.06
Relative protein	0.01 (0.005)	0.05
Relative fibre	−0.12 (0.03)	<0.001
Mean foods per CE	−0.11 (0.05)	0.02
Variability foods per CE	0.01 (0.08)	0.87
Variability time gap	0.01 (0.003)	0.004
IDD first CE	0.01 (0.08)	0.92
IDD number of CEs	0.02 (0.12)	0.86

## Data Availability

The NHANES 2013-4 data are downloadable from https://wwwn.cdc.gov/nchs/nhanes/Default.aspx. The R code required to reproduce all our analyses, or perform other analyses with the derived variables we created, is freely available on the Zenodo repository at: https://doi.org/10.5281/zenodo.2649031. The repository submission includes information on which NHANES files are required.

## Supplementary Materials

Supplementary Materials Table S1: women's estimated marginal means and standard errors by the food insecurity category, using the four-level classification of the USDA score rather than the dichotomous classification of food-secure versus food-insecure. Table S2: parameter estimates for the difference between food-secure and food-insecure men. Adjusted models include age, income, education, ethnicity, and presence of children in the household as additional predictors. Food-secure is the reference category, and hence the parameter estimates represent the deviation of food-insecure men from the food-secure mean. Table S3: results of models testing whether each of the food consumption variables significantly associated with food insecurity predicts body mass index in NHANES men. All models are adjusted for age, income, education, ethnicity, and presence of children in the household. Figure S1: forest plot of standardized associations between food insecurity status and food consumption variables for NHANES men after adjustment for age, income, education, ethnicity, and presence of children in the household. Variables are sorted so that those more strongly associated with food insecurity status appear higher on the figure. A negative value indicates that food-insecure women have a lower value of the parameter, and a positive value a higher value. Whiskers represent 95% confidence intervals. CE: consumption event. IDD: interday difference (for participants with two separate days of recall data).
