# Supplementary material for: Food-Insecure Women Eat a Less Diverse Diet in a More Temporally Variable Way: Evidence from the US National Health and Nutrition Examination Survey, 2013-4
Source: J Obes. 2019 Oct 1;2019:7174058. doi: 10.1155/2019/7174058 (PMC6791191; doi:10.1155/2019/7174058)
Supplement: Supplementary Materials — Table S1: women's estimated marginal means and standard errors by the food insecurity category, using the four-level classification of the USDA score rather than the dichotomous classification of food-secure versus food-insecure. Table S2: parameter estimates for the difference between food-secure and food-insecure men. Adjusted models include age, income, education, ethnicity, and presence of children in the household as additional predictors. Food-secure is the reference category, and hence the parameter estimates represent the deviation of food-insecure men from the food-secure mean. Table S3: results of models testing whether each of the food consumption variables significantly associated with food insecurity predicts body mass index in NHANES men. All models are adjusted for age, income, education, ethnicity, and presence of children in the household. Figure S1: forest plot of standardized associations between food insecurity status and food consumption variables for NHANES men after adjustment for age, income, education, ethnicity, and presence of children in the household. Variables are sorted so that those more strongly associated with food insecurity status appear higher on the figure. A negative value indicates that food-insecure women have a lower value of the parameter, and a positive value a higher value. Whiskers represent 95% confidence intervals. CE: consumption event. IDD: interday difference (for participants with two separate days of recall data). [file 7174058.f1.pdf]

Supplementary Materials for Nettle & Bateson 'Food-insecure women eat a less diverse diet in a more temporally variable way: Evidence from the US National Health and Nutrition Examination Survey, 2013-4': Additional analyses

Section 1: Analyses by severity of FI

For the eight significant differences between food-secure and food-insecure women reported in the right-hand columns of table 2, we repeated the adjusted model using the four-level classification of food insecurity status defined in the USDA questionnaire manual instead of the food secure/food insecure dichotomy. The four levels were: 1. Full food security (score of 0; identical to 'food secure' in our dichotomous classification, n = 1895); 2. Marginal food security (score of 1 or 2; n = 322); 3. Low food security (score of 3-5; n = 335); and 4. Very low food security (score of 6-10, n = 235). In all eight cases, FI category was a highly significant predictor of the dietary outcome variable, even after adjustment (table S1). The estimated marginal means by level of food insecurity general showed evidence of a severity gradient: the fully food secure and very low food security group typically had the most divergent means, with the other two groups falling in the middle (table S1). Significant pairwise group differences were most often found between the two extreme groups, though it must be noted that, given the number of control variables, the relatively small numbers of women in the food-insecure groups, and correction for multiple testing, power to detect significant pairwise group differences was modest.

Table S1. Women's estimated marginal means and standard errors by food insecurity category, using the four-level classification of the USDA score rather than the dichotomous classification of food secure versus food insecure.

Variable	Overall effect of FI	1: Fully food-secure	2: Marginally food-secure	3: Low food-security	4: Very low food-security
Relative carbohydrate	$F_{3, 2581}$ = 24.26, $p < 0.001$	1.85 (1.13)[4]	3.84 (2.08) [4]	3.98 (2.06) [4]	13.03 (2.45) [1,2,3]
Relative protein	$F_{3, 2581}$ = 12.73, $p < 0.001$	-1.51 (0.76) [4]	-3.36 (1.40)	-2.66 (1.39)	-7.59 (1.65) [1]
Relative fibre	$F_{3, 2581}$ = 23.49, $p < 0.001$	1.12 (0.22) [4]	0.63 (0.41)	0.33 (0.41)	-0.17 (0.49) [1]
Mean foods per CE	$F_{3, 2581}$ = 44.15, $p < 0.001$	9.77 (0.11)[4]	9.54 (0.21)	9.29 (0.21)	9.12 (0.25)[1]
Variability foods per CE	$F_{3, 2564}$ = 44.28, $p < 0.001$	5.42 (0.07)[4]	5.20 (0.13)	5.21 (0.13)	4.91 (0.15)[1]
Variability time gap	$F_{3, 2485}$ = 24.74, $p < 0.001$	102 (1.73)[4]	110 (3.22)	107 (3.20)	119 (3.77)[1]
IDD first CE	$F_{3, 2346}$ = 13.75, $p < 0.001$	1.57 (0.08)[2,3]	2.06 (0.15)[1, 4]	1.97 (0.15)[1, 4]	1.34 (0.17)[2,3]
IDD number of CEs	$F_{3, 2346}$ = 4.14, $p = 0.006$	1.45 (0.05)[3]	1.41 (0.10)	1.73 (0.10)[1]	1.71 (0.11)

Notes: All models are adjusted for age, income, education, ethnicity, and presence of children in the household. F-ratios test for the overall effect of FI category. Superscript numbers after the estimated marginal means indicate which other categories the mean is significantly different from ($p < 0.05$) using Tukey pairwise multiple comparisons.

Section 2: Analyses of data from male participants

We repeated the analyses conducted on the female data on the data from the male participants from the NHANES 2013-4 cycle (n = 2558; 2208 with 2 days of food recall data, 350 with 1 day). Table S2 presents the parallel analyses to table 2 for the men, and figure S1 shows the male equivalent of figure 1.

Results were in many respects similar for the men as for the women. All three MANOVAs showed significant differences between food-insecure and food-secure men, both adjusted and unadjusted. The individual variables showing strong directional differences in the women also tended to do so in the men (correlations, across variables, between male and female standardized effect sizes, r = 0.66, p < 0.001). Of the eight individual variables showing a significant association with FI after adjustment in the women, five also did so in the men (relative carbohydrate, relative protein, relative fibre, mean foods per CE, variability foods per CE). These associations were all in the same direction in the two genders. The remaining three variables that showed a significant difference by FI in the women showed no significant difference by FI in the men (variability time gap, IDD first CE, IDD number of CEs). One of these (variability time gap) was only marginally non-significant, and in the same direction as in the women. Thus, the pattern of differences in food consumption variables between food-insecure and food-secure individuals appears similar across men and women. We have also confirmed that FI affects both genders similarly by combining the male and female data and running models with FI, gender and their interaction as predictors. We generally found main effects of FI, rather than interactions between FI and gender (data not shown).

We confirmed that FI does not predict BMI in the men, either in unadjusted (B = 0.007, se 0.28, p = 0.98) or adjusted (B = 0.14, se 0.33, p = 0.66) analyses.

We repeated the analyses of table 3 of the main paper – that is, analyses of which food consumption variables predicted BMI – for the men (table S3). There were actually more food consumption variables significantly associated with BMI for men than women (five significant associations for men versus four for women), and generally the same ones in the same directions (women: relative protein, relative fibre, mean foods per CE, variability time gap; men: relative carbohydrate, relative protein, relative fibre, mean foods per CE, variability foods per CE). Variability in time gap was not significantly associated with BMI in the men, whereas it was one of the mediating variables in the women. Thus, of the three food consumption variables that partially mediate the FI-BMI association in the women (relative fibre consumption, mean foods per CE, variability time gap), two (relative fibre consumption and mean foods per CE) are associated both with FI and with BMI in men, just as they are in women. The only difference is that they cannot serve as statistical mediators, since there is no overall association to mediate. The third (variability in time gap), which was a partial mediator in women, was marginally non-significantly associated with FI, and non-significantly associated with BMI, in the men.

Table S2. Parameter estimates for the difference between food-secure and food-insecure men. Adjusted models include age, income, education, ethnicity, and presence of children in the household as additional predictors. Food secure is the reference category and hence the parameter estimates represent the deviation of food-insecure men from the food-secure mean.

	Unadjusted		Adjusted	
	B (se)	p-value	B (se)	p-value
Consumption variables	*MANOVA F(5, 2552) = 19.46*	*< 0.001*	*MANOVA F(5, 2351) = 20.26*	*< 0.001*
Energy intake	135.37 (43.55)	0.002	51.10 (51.10)	0.32
Relative carbohydrate	12.42 (1.96)	<0.001	6.92 (2.31)	0.003
Relative protein	-5.53 (1.41)	<0.001	-4.07 (1.66)	0.01
Relative fat	-4.15 (1.12)	<0.001	-1.59 (1.31)	0.22
Relative fibre	-2.74 (0.37)	< 0.001	-1.26 (0.41)	0.002
Intra-day pattern variables	*MANOVA F(6, 2416) = 20.96*	*< 0.001*	*MANOVA F(6, 2228) = 22.75*	*< 0.001*
First CE	0.45 (0.11)	<0.001	0.12 (0.13)	0.36
Number of CEs	-0.49 (0.08)	<0.001	-0.14 (0.09)	0.14
Mean foods per CE	-1.61 (0.16)	<0.001	-0.38 (0.17)	0.03
Variability foods per CE	-0.94 (0.09)	<0.001	-0.28 (0.10)	0.006
Variability time gap	13.58 (2.79)	<0.001	6.58 (3.33)	0.05
Variability energy per CE	34.74 (9.72)	<0.001	3.44 (11.51)	0.76
Inter-day variability variables	*MANOVA F(5, 2186) = 11.02*	*<0.001*	*MANOVA F(5, 2022) = 10.11*	*<0.001*
IDD energy intake	189.12 (37.41)	<0.001	80.27 (44.05)	0.07
IDD first CE	0.36 (0.13)	0.008	-0.12 (0.16)	0.45
IDD number of foods	-0.004 (0.21)	0.99	0.008 (0.25)	0.98
IDD number of CEs	-0.01 (0.07)	0.83	-0.07 (0.08)	0.42
IDD mean time gap	17.61 (3.74)	<0.001	2.69 (4.44)	0.55

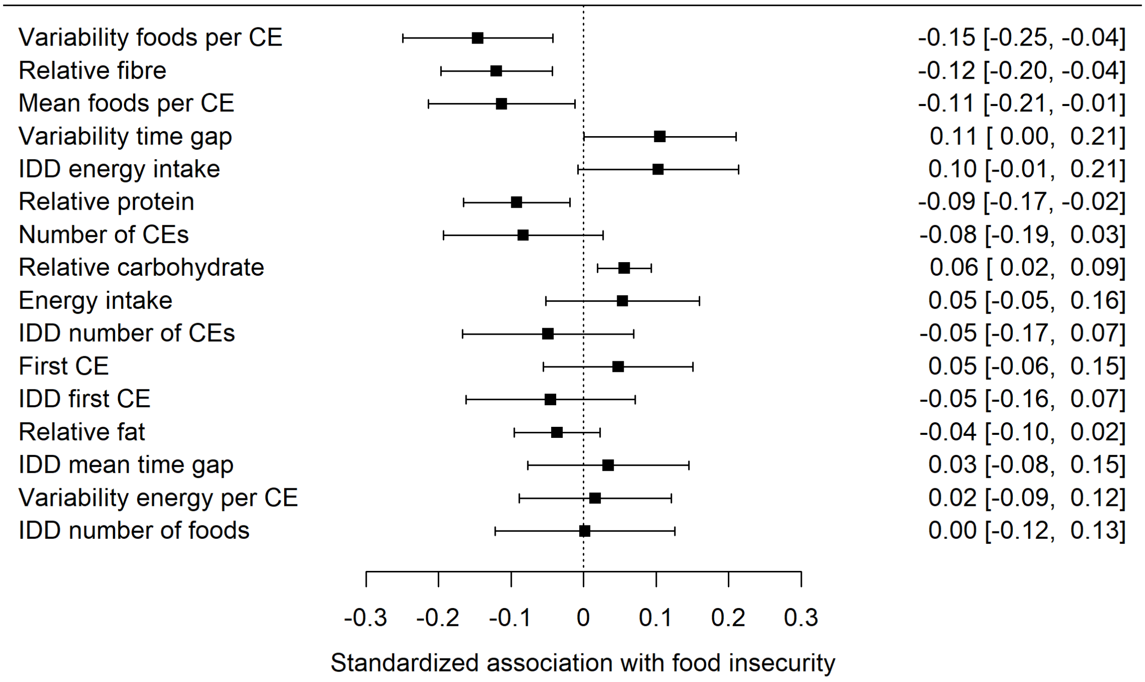

Figure S1. Forest plot of standardized associations between food insecurity status and food consumption variables for NHANES men, after adjustment for age, income, education, ethnicity, and presence of children in the household. Variables are sorted so that those more strongly associated with food insecurity status appear higher on the figure. A negative value indicates that food-insecure women have a lower value of the parameter, and a positive value a higher value. Whiskers represent 95% confidence intervals. CE: Consumption event. IDD: Inter-day difference (for participants with two separate days of recall data).

Table S3. Results of models testing whether each of the food consumption variables significantly associated with food insecurity predicts body mass index in NHANES men. All models are adjusted for age, income, education, ethnicity, and presence of children in the household.

Predictor	B (se)	p-value
Relative carbohydrate	-0.01 (0.003)	0.002
Relative protein	0.01 (0.004)	0.02
Relative fibre	-0.04 (0.02)	0.03
Mean foods per CE	-0.27 (0.04)	<0.001
Variability foods per CE	-0.26 (0.07)	<0.001
Variability time gap	0.002 (0.002)	0.29
IDD first CE	-0.003 (0.05)	0.95
IDD number of CEs	0.003 (0.10)	0.98
